# Editorial: Seed-environment interactions

**DOI:** 10.3389/fpls.2023.1201047

**Published:** 2023-05-04

**Authors:** Fei Xu, Muhammad Ahsan Asghar

**Affiliations:** ^1^ Applied Biotechnology Center, Wuhan University of Bioengineering, Wuhan, China; ^2^ Department of Biological Resources, Agricultural Institute, Centre for Agricultural Research, ELKH, Martonvásár, Hungary

**Keywords:** seed physiology, stress acclimation, environmental factors, germination, molecular mechanism

Seeds represent the main assets for nature-based solutions for species propagation, they are the link between the end of the reproductive cycle of adult plants and the establishment of their next generation, with huge implications for their succession, regeneration, and conservation that are driven by seed dispersal. Seed development, maturation, storage, and germination are complex physiological events, strongly determined by the internal factors (e.g., seed maturity and vigor, bacterial and fungal endophytes, etc.) (Vivas et al., 2013; Kang et al., 2016; Berg and Raaijmakers, 2018; Lamichhane et al., 2018), which interact with environmental conditions (e.g., temperature, humidity, pathogens, etc.) in order to identify the best timing for germination. With the change of global climate, including drought and water shortage, high temperature or extreme cold, acid rain, ozone and ultraviolet radiation, etc. (Li et al., 2011; Leisner et al., 2017; Lamichhane et al., 2018; Nguyen et al., 2021; Vancostenoble et al., 2022), the soil environment has also undergone great changes, such as the increase of saline-alkali land in the north, and the severe acidification of the soil in parts of the south (Footitt and Cohn, 1992; Olsson and Kellner, 2002; Roem et al., 2002; Lowell et al., 2021). In this context, what physiological and molecular changes occur or what coping strategies are activated during the interaction of seeds with the environment, as seeds are self-sufficient biological entities capable of persevering in harsh environments? Therefore, it is imperative to understand the basis of seed-environment interaction which is very critical for sustainable agriculture.

In this topic, several articles studied the influence of environmental factors such as temperature, water potential, and oxygen partial pressure on seed germination and vitality. Among them, Bao et al. expanded our knowledge of the potential effect of temperature and water potential on seed germination and vigor and the subsequent seedling establishment of five populations of *Pedicularis kansuensis* in cool and warm habitats. Based on thermal time and hydrotime models, the seed germination of the targeted weed was assessed. The results indicated that the seeds of cool habitats had a greater base temperature as compared to the seeds of warm habitats. In addition, it should be noted that the seed germinability was different among the tested populations in response to water potential. A negative correlation was noticed between the seed populations’ base water potential for 50% (Ψb_(50)_) germination and their hydrotime constant (qH). In conclusion the authors proposed that the thermal time and hydrotime models could be good predictors in determining the seed germination rate and its vigor in different plant species.

In another study, the seed dormancy status was evaluated under the stratification of different temperature regimes (Zhou et al.). Notably, when the seeds were stratified with autumn and spring temperature regimes, the greater embryo length, embryo-to-seed ratio, and seed percentage were noticed as compared to winter and summer temperatures’ stratifications. It was also found that the warm temperatures promoted embryo growth, and the cold temperatures were responsible for breaking the physiological dormancy of the *T. chinensis* var. mairei seeds. Interestingly, in the study of Lee et al., the dormancy class and breaking method of *Amsonia elliptica* (Apocynaceae) were investigated by using the wet stratified condition. It was found that among the stratifications (cold moist stratification and warm moist stratification), the pretreatment of warm stratification before cold stratification was more effective in breaking the dormancy and hence improving the seed germination rate. The authors further observed that soaking of *Amsonia elliptica* seeds in 500 mg/L GA4 + 7 for 14 days resulted in the efficient breaking of the dormancy that led to improved seed growth as compared to warm plus cold stratification treatments. Henceforth, it was concluded that the methods of this study can be useful for the restoration of *Amsonia elliptica* species which will empower the horticultural and medicinal industries.

It is well known that seed dormancy and germination are regulated by plant hormones. Research findings from this topic have shown that environmental factors such as temperature and plant endogenous hormones can jointly affect seed germination. Zhou et al. found that abscisic acid (ABA) levels substantially declined during the stratification of *T. chinensis* var. *mairei* seeds at the simulated seasonal temperature regimes. However, no significant changes were observed for indole acetic acid (IAA) and gibberellic acid (GA). Interestingly, the IAA–ABA and GA–ABA ratios enhanced during all the simulated temperatures being the highest following spring temperature stratification. Taken altogether, this study broadens our knowledge that the morphophysiological dormancy of the *T. chinensis* var. mairei seeds was largely regulated by the balance of endogenous hormones and environmental factors.

The potential use of new biotechnological methods in seed aging will have strong application value in future agriculture, as these methods will allow direct intervention in the storage and germination of any seed without changing its genetic background. The effects of elevated partial pressure of oxygen (EPPO) (200 times higher oxygen levels) under different conditions were investigated on rice seeds (20 diverse accessions) during storage (Prasad et al.). The authors noticed substantial aging of rice seeds stored under EPPO which was accompanied by a lower germination rate. The high-pressure nitrogen gas also resulted in a substantial decline in the germination and vigor of rice seeds. In another experiment, an untargeted metabolomics approach was used to investigate the effect of different storage conditions, i.e., EPPO, moist-controlled deterioration, conditioned warehouse seed storage, and traditional rice seed storage. Interestingly, a majority of metabolites were upregulated under all the aforementioned storage conditions. Furthermore, these metabolites showed a strong negative relationship with seed viability. The EPPO method was concluded to be an effective way to artificially induce seed aging to enable comparative studies between different seed batches and genotypes in rice.

Overall, this Research Topic provides deeper insights into the interaction of seeds with the environment and highlights some of the important factors contributing to seed germination, vigor, and viability. Since the tested crops are very economically important, this Research Topic emphasizes the pressing need for improving the seed performance and the subsequent seedling establishment by various dormancy-breaking methods. We hope that this special focus issue on the relationship between seeds and the environment will serve as an important reference for the interaction between seeds and their biotic and abiotic environment.

## Author contributions

FX: conceptualization. MA: writing—original draft preparation. All authors listed have made a substantial, direct, and intellectual contribution to reviewing and editing this version of the work and approved it for publication.
